# The genomics of rapid climatic adaptation and parallel evolution in North American house mice

**DOI:** 10.1371/journal.pgen.1009495

**Published:** 2021-04-29

**Authors:** Kathleen G. Ferris, Andreas S. Chavez, Taichi A. Suzuki, Elizabeth J. Beckman, Megan Phifer-Rixey, Ke Bi, Michael W. Nachman

**Affiliations:** Museum of Vertebrate Zoology and Department of Integrative Biology, University of California Berkeley, Berkeley, California, United States of America; University of Pennsylvania, UNITED STATES

## Abstract

Parallel changes in genotype and phenotype in response to similar selection pressures in different populations provide compelling evidence of adaptation. House mice (*Mus musculus domesticus*) have recently colonized North America and are found in a wide range of environments. Here we measure phenotypic and genotypic differentiation among house mice from five populations sampled across 21° of latitude in western North America, and we compare our results to a parallel latitudinal cline in eastern North America. First, we show that mice are genetically differentiated between transects, indicating that they have independently colonized similar environments in eastern and western North America. Next, we find genetically-based differences in body weight and nest building behavior between mice from the ends of the western transect which mirror differences seen in the eastern transect, demonstrating parallel phenotypic change. We then conduct genome-wide scans for selection and a genome-wide association study to identify targets of selection and candidate genes for body weight. We find some genomic signatures that are unique to each transect, indicating population-specific responses to selection. However, there is significant overlap between genes under selection in eastern and western house mouse transects, providing evidence of parallel genetic evolution in response to similar selection pressures across North America.

## Author summary

Dissecting the genetic basis of parallel evolution, the independent evolution of similar phenotypes in similar environments among closely related lineages, allows evolutionary biologists to test whether evolution is predictable at the molecular level. Relatively little is still known about the genetics of parallel evolution in quantitative traits. Here we identify significant phenotypic and genomic parallel evolution in quantitative traits across two latitudinal transects of wild house mice in eastern and western North America. We find parallel evolution in thermally adaptive phenotypes (nest building behavior and body mass) and in genes involved in temperature-related traits such as body mass, metabolism, and temperature-sensing using population genomic scans for selection. We also find considerable divergent phenotypic and genomic evolution between eastern and western transects corresponding to known environmental differences between these transects. In this case, the evolution of quantitative traits across similar latitudinal transects involved a mixture of unique and shared responses to selection at the molecular level.

## Introduction

A central goal of evolutionary biology is to understand how organisms adapt to novel environments. The geographic distribution of genotypes and phenotypes can provide information about the targets of spatially varying selection [1,2]. For example, clinal patterns of variation in *Drosophila* have been described for individual genes [3–5] and various traits such as fecundity [6] and wing size [7]. More recent work in *Drosophila* has taken a genome-wide approach, which has the advantage of being agnostic with respect to phenotype and thus has the potential to identify previously unsuspected targets of selection [8–11]. Genome-wide surveys of clinal variation have also been adopted in many other organisms [12–19].

To understand the predictability of adaptive evolution in response to spatial variation in selection pressures, several studies have looked for repeated patterns of evolution across different populations that have experienced similar environments and selection pressures. For example, sticklebacks have repeatedly colonized freshwater lakes and streams from marine environments. Comparison of pairs of freshwater and marine populations has led to the discovery of genes that show parallel changes in independent transects [15]. Similarly, *Drosophila melanogaster* has independently colonized Australia and North America from its ancestral range. Consistent latitudinal clines of genetic and phenotypic variation have been identified on both continents [8,9]. Parallel patterns of clinal variation across independent transects provide strong evidence that the traits in question are adaptive. In cases where parallel phenotypic clines are observed, the discovery of parallel genetic clines illustrates the repeatability of evolution at the molecular level. For example, in sticklebacks, most freshwater populations share a suite of common phenotypic changes. Genetic patterns of variation reveal both parallel clines and some transect-specific clines, suggesting that adaptation to a freshwater environment may involve a mix of both shared and unique genetic changes [15,20].

Despite this extensive previous work, links between genotype and phenotype are still relatively uncommon in the context of clinal variation. Notable exceptions include protein variants such as *Adh* in *Drosophila* [4,21], *Pgi* in butterflies [22–24], and *Ldh* in killifish [25,26]. In some cases, the genetic basis of clinally varying phenotypes has been identified through traditional mapping strategies [5,27]. One of the challenges of identifying the genes underlying clinal phenotypic variation is that many traits are highly polygenic with only modest contributions from individual genes. While there have been notable successes in identifying the genetic basis of parallel evolution in Mendelian or oligogenic traits such as cardenolide resistance [28], coat color [29–31], or body armor in sticklebacks [27], much less is known about the extent of genetic parallelism in quantitative traits. In principle, one might expect highly polygenic traits to show less genetic parallelism since there may be numerous genetic paths to the same phenotypic optimum. One approach to identifying the genomic extent of parallel evolution in polygenic traits is to combine population genomic scans for selection across independent environmental clines [32], measurement of complex phenotypes in a common environment [33], and genome-wide association studies (GWAS) of the traits of interest [34].

The recent introduction of house mice, *Mus musculus domesticus*, into the Americas from Western Europe provides an opportunity to study rapid and parallel environmental adaptation. In their native range in Western Europe, house mice live in temperate climates. However, since their introduction into the Americas, *M*. *m*. *domesticus* have expanded into many novel environments from Alaska to the tip of South America, including the subarctic, xeric, and tropical climatic zones [35,36]. Throughout this range house mice frequently occupy outdoor structures, such as barns and sheds, exposing them to greater environmental variation than that which is experienced by humans. This recent population expansion into new and extreme habitats, combined with their status as a mammalian model system, makes house mice useful for studying the genetic basis of rapid local adaptation. Additionally, their colonization of multiple similar thermal environments across the Americas provides a powerful system to study the genomic basis of parallel evolution in response to temperature.

**Fig 1:**
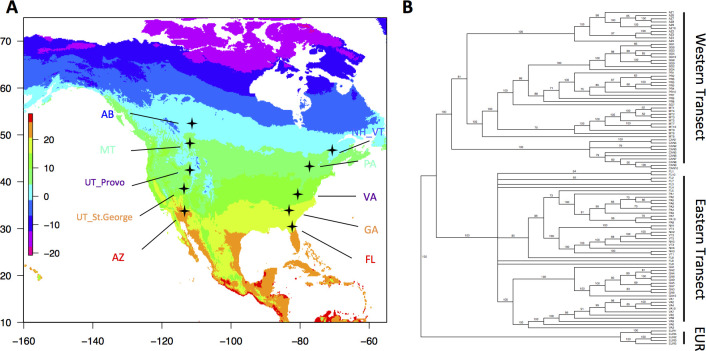
Sampling localities and relationships among populations. A) Heat map of mean annual temperature (MAT) in North America with the parallel eastern and western house mouse transects with populations at similar latitudes shown in the same color. Degrees of latitude are marked on the y-axis, longitude on the x-axis, and MAT in °C is indicated by color. *This map was created using the R package “raster’ to plot Mean Annual Temperature data from worldclim (MAT = bioclim variable 1)*. B) A bootstrap consensus neighbor-joining tree constructed in PAUP using a distance matrix generated from the exome sequences of all 100 mice in both the eastern and western transects depicting the relatedness among all 10 populations. This tree is rooted using five *M*. *m*. *domesticus* samples from Europe.

In this study we examine rapid adaptation along a latitudinal cline in western North America and compare our results to a previous study of clinal variation in house mice along a similar thermal gradient in eastern North America (Fig 1A) [33]. We analyzed the phenotypic and genomic basis of adaptation using wild mice collected from five populations between Tucson, Arizona and Edmonton, Alberta. The environment varies dramatically in temperature, precipitation, and seasonality across this latitudinal transect (S1 Fig). We first measured body weight and nest-building behavior, traits involved in thermal adaptation, in lab-reared descendants of wild mice collected from the ends of the western transect. We then performed a population genomic scan for selection and a genome wide association study on body weight using exome data to identify candidate loci for adaptive variation in quantitative traits in the western transect. Finally, we examined the degree of parallel genomic evolution by determining the overlap between the loci under selection in eastern and western North America. While both transects traverse a similar thermal range, the eastern transect does not share the western transect’s striking precipitation gradient suggesting there will be both strong parallel and non-parallel selective forces driving clinal variation in house mice across North America (S1 Fig) [37].

## Results

### Independent evolutionary history of eastern and western populations

Parallel phenotypic and genetic clines in eastern and western North America could result from independent evolution or shared history among populations at the same latitudes. To distinguish between these possibilities, we reconstructed the history of the 10 populations in both transects using exome data generated here from 50 wild mice across 21° of latitude in western North America (Fig 1A, collecting localities and samples are given in S1 Table, and exome data are summarized in S2 and S3 Tables), and exome data from the five eastern populations in [33]. Unrelated wild-caught mice were sampled in the same way in both transects, with 10 mice per locality. A neighbor-joining tree generated from a distance matrix based on the exome sequences of mice in both transects depicts the relationships among these 10 populations using mice from the ancestral European range to root the tree (Fig 1B). In general, mice within populations are more closely related to each other than they are to mice from other populations (Fig 1B). At a larger geographic scale, mice within each transect are more closely related to each other than they are to mice from the other transect. The tree contains two major clades, one containing mice from western North America and one containing mice from eastern North America. Both of these clades have a bootstrap support of 100%. We also generated a population tree using quartet assembly with random sampling of 50,000 quartets with SVDQuartets (see Materials and Methods). This tree also depicts two major clades corresponding to the eastern and western transects, with 100% bootstrap support for each clade (S2 Fig). Similar patterns can be seen in a principal components analysis, in which mice from each transect are separated along PC1 (S3 Fig). Thus, populations are clustered broadly by longitude rather than by latitude, suggesting that adaptation to different latitudes likely occurred independently in these two transects. An alternative possibility is that adaptation to high latitudes occurred once and that beneficial alleles were carried by rare migrants between the eastern and western transects. This seems less likely in light of the very recent colonization of house mice in the Americas and the well-supported phylogenetic relationships depicted in Figs 1B and S2.

**Fig 2:**
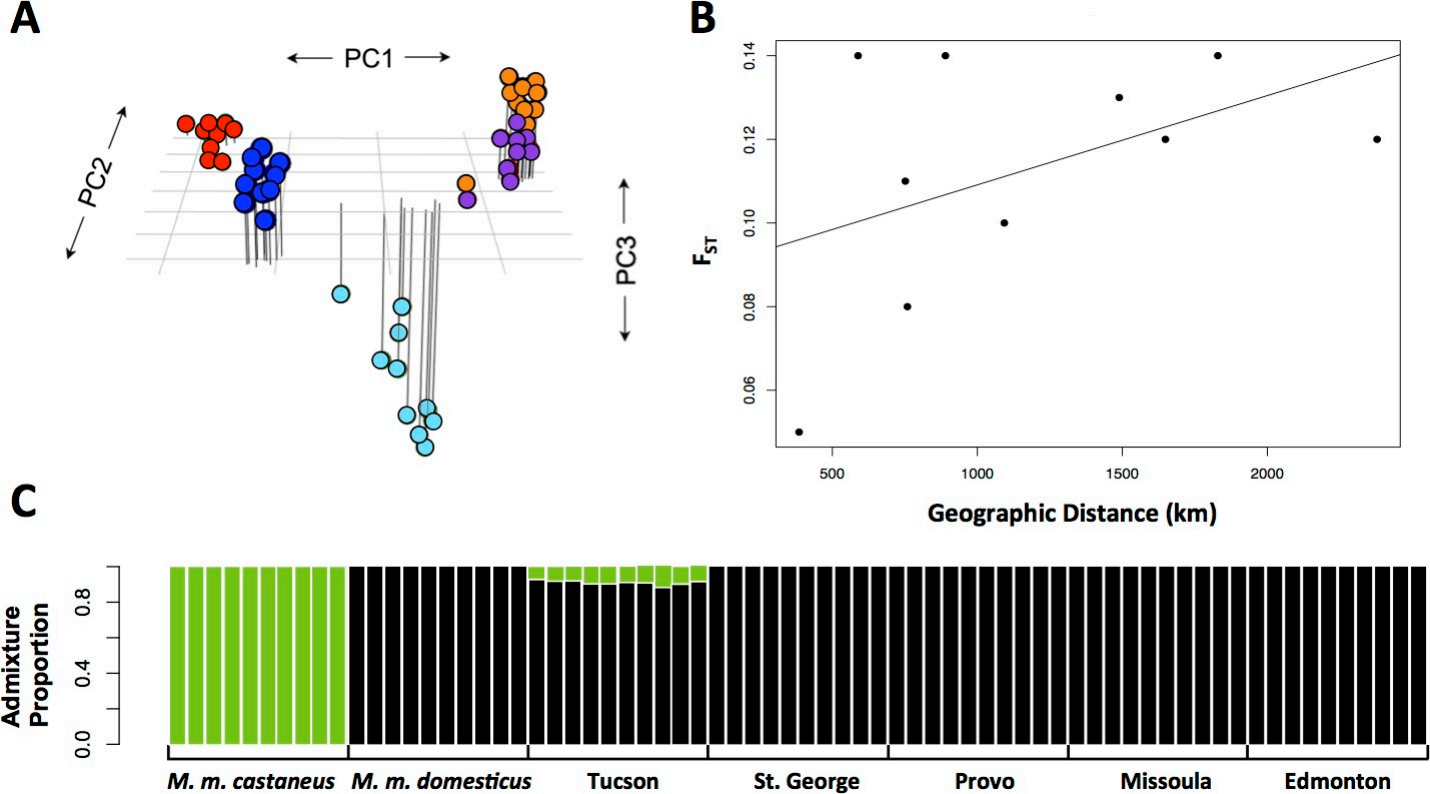
Population structure in mice from the western transect. A) Three dimensional principal components plot of the five western populations from Fig 1: Tucson, AZ (red), St. George, UT (orange), Provo, UT (purple), Missoula, MT (cyran), and Edmonton, AB (dark blue); B) plot of genetic distance as measured by Fst versus geographic distance (km); C) admixture plot of the five western populations with *M*. *m*. *castaneus* ancestry plotted in green and *M*. *m*. *domesticus* ancestry plotted in black.

We also documented genome-wide patterns of genetic variation within western North America. Overall levels of nucleotide diversity (π) within populations ranged from 0.14% to 0.25% (S4 Table), similar to levels of variation seen in the eastern transect [33]. Also similar to patterns observed in the eastern transect, principal components analysis largely grouped western mice by population (Fig 2A), which is consistent with the neighbor-joining tree depicted in Fig 1B. We observed a modest signature of isolation by distance (IBD) in the western transect (R^2^ = 0.28, Fig 2B), in contrast to the lack of IBD found in the eastern transect [33].

To better understand the subspecific origin of house mice in the western transect we used the software ngsAdmix to test for admixture between *M*. *m*. *castaneus* and *M*. *m*. *domesticus*. We detected a small signature of admixture between *M*. *m*. *castaneus* and *M*. *m*. *domesticus* in the Tucson, AZ population, but no evidence of admixture in the other four populations (Fig 2C). No signature of admixture among house mouse subspecies was found in the eastern transect [33]. Admixture in the Tucson population may explain the finding that this population has the highest pairwise F_ST_ (mean = 0.13) and sequence diversity (π = 0.0025, θ_ω_ = 0.0024) of any population in the western transect (S4 and S5 Tables).

### Phenotypic variation in house mice from western North America

**Fig 3:**
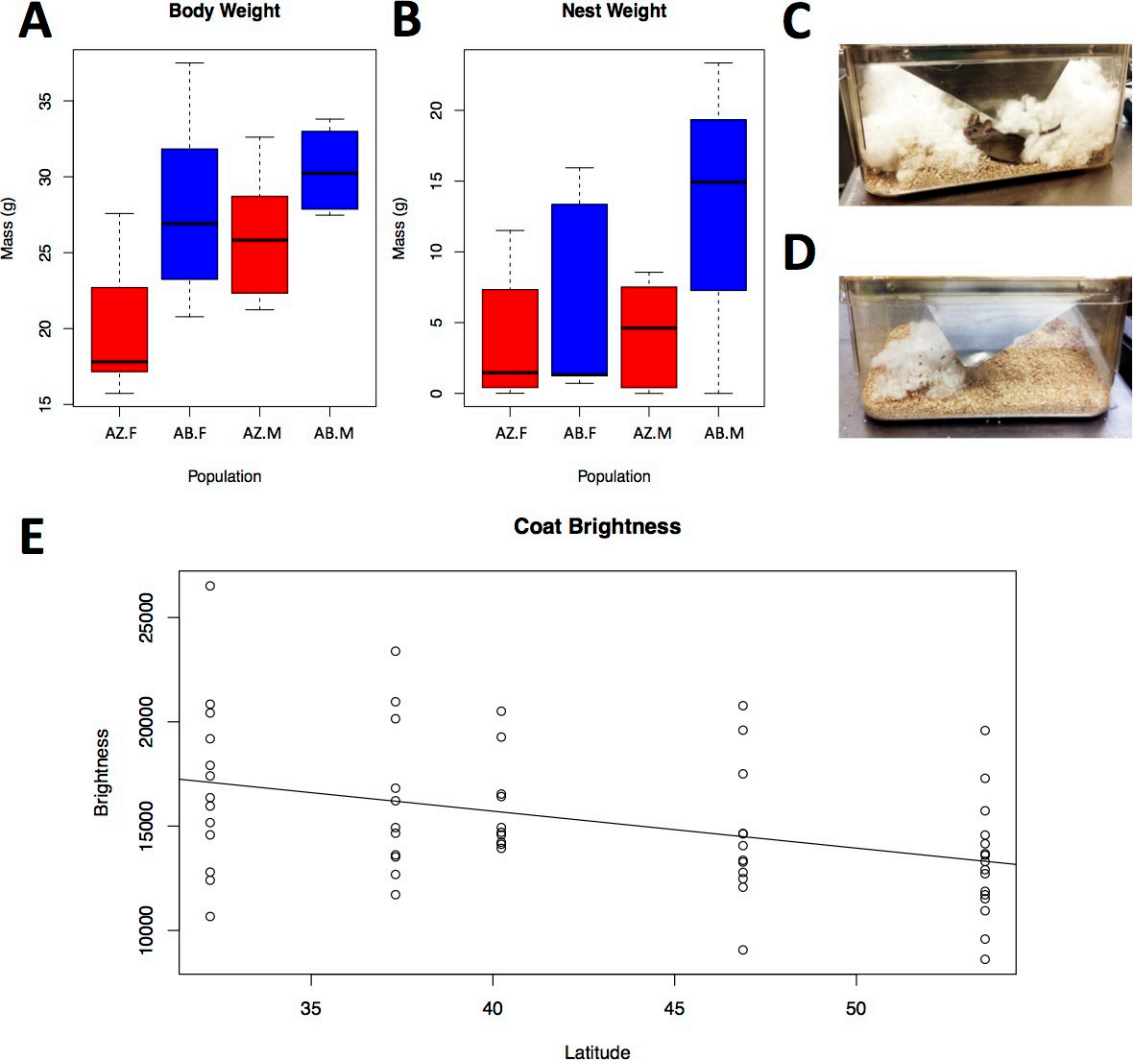
Phenotypic variation in lab-reared and wild-caught mice. A) Body mass of fifth-generation laboratory-reared female (F) and male (M) mice from Arizona (red) and Alberta (blue); B) mass of nesting material used by fifth-generation laboratory-reared female (F) and male (M) mice from Arizona (red) and Alberta (blue) in a 24-hour period; C) a typical large nest built by a mouse from Alberta; D) a typical small nest built by a mouse from Arizona; E) brightness of the dorsal fur of 50 wild-caught mice as measured by a spectrometer. Brightness is measured as the total area under the average reflectance curve from 300–700 nm in arbitrary units.

Next, we sought to characterize potentially adaptive phenotypic variation in the western transect and compare this to previously documented variation in the eastern transect. We focused first on body weight and nest-building behavior (see Materials and Methods), two traits involved in thermal adaptation and known to differ between northern and southern populations in the eastern transect [33]. Among fifth-generation lab-born descendants of wild-caught mice, we found that body mass was significantly greater in Alberta mice than in Arizona mice (ANOVA; population p = 0.003, sex p = 0.035, age p = 0.547 Fig 3A). We also found that nest weight was significantly greater in Alberta mice than in Arizona mice (ANOVA; population p = 0.038, body weight p = 0.966, sex p = 0.394, age p = 0.954; Fig 3B, 3C, and 3D). The magnitude and direction of these differences were similar to those seen in the eastern transect [33]. The fact that these differences persisted over multiple generations among descendants of wild-caught animals reared in a common laboratory environment indicates that the differences have a genetic basis and are not due to either phenotypic plasticity or maternal effects. These genetic differences indicate that there has been parallel phenotypic evolution in morphology and behavior between eastern and western transects.

We also characterized phenotypic variation among wild-caught animals. These animals likely varied in age, health, reproductive status, pathogen exposure, diet and many other unknown variables. We did not observe a significant latitudinal cline for body weight (p = 0.573). Since we did see significant differences in body weight in the lab reared mice, the lack of a significant cline among wild mice is likely due to the relatively small sample sizes per population as well as the influence of uncontrolled factors such as age, diet, and health. However, we did observe a significant cline for coat color in terms of brightness (Fig 3E; p = 0.001). Darker mice were observed in more northern latitudes where darker soils are associated with more humid environments and higher degrees of organic matter in the soil. In the western transect, this variation in coat color was readily discernable by eye. In contrast, no discernable variation in coat color was observed in the eastern transect, and therefore spectrophotometric data were not collected from eastern mice.

### The genomic signature of adaptation in mice from western North America

**Fig 4:**
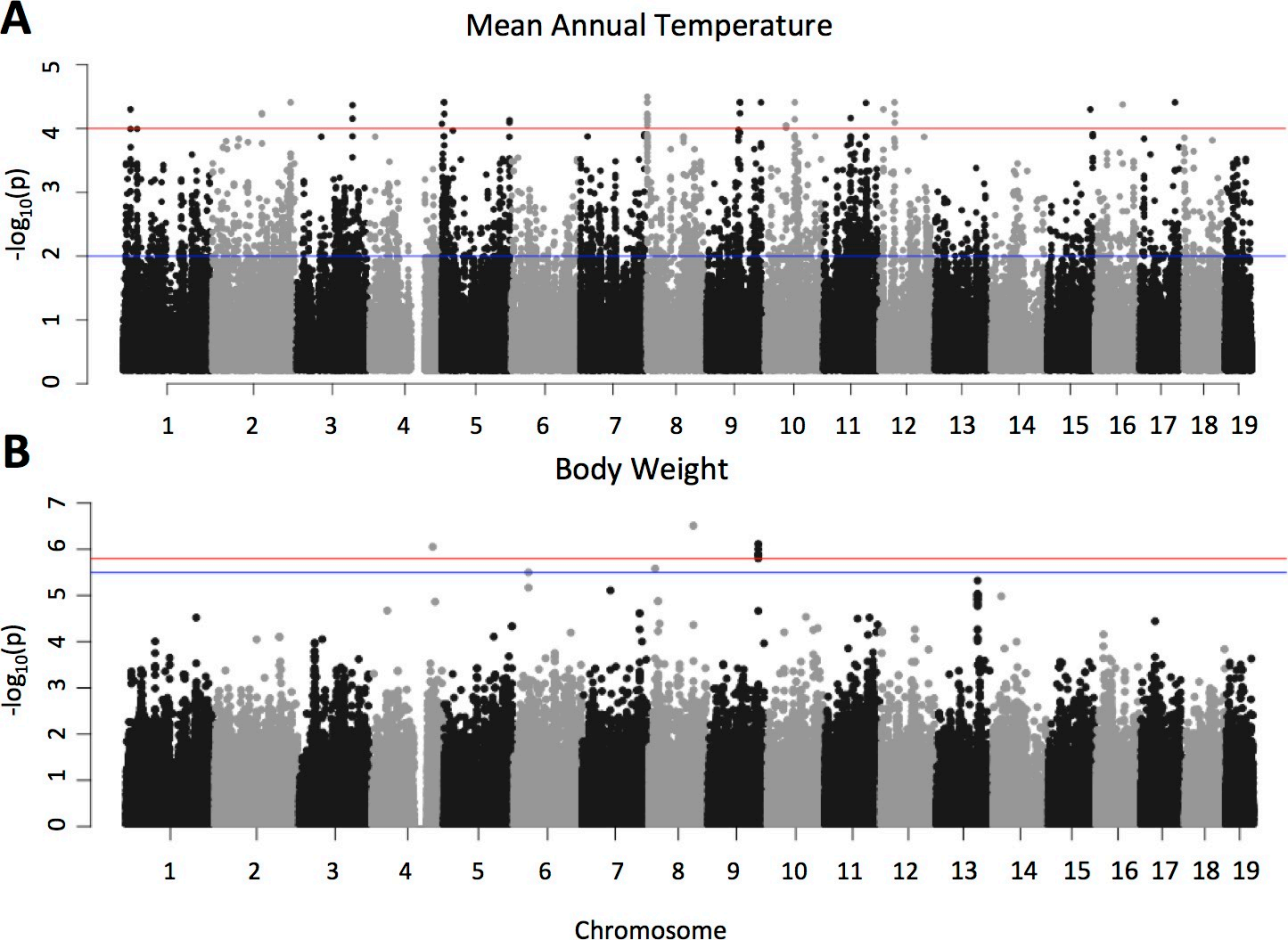
Manhattan plots depicting the results of A) a population genomic scan for selection using LFMM (blue line indicates q-value = 0.05 and the red line indicates q-value = 0.001; B) a genome-wide association study of body weight using 38 mice from the western transect (blue line indicates a q-value = 0.01 and the red line indicates a q-value = 0.0001).

To examine regions of the genome contributing to environmental adaption across western North America, we performed population genomic scans for selection using the latent factor mixed model (LFMM) method [38]. With a q-value cut-off of 0.05 we identified 13,057 single nucleotide polymorphisms (SNPs) in 4438 genes that were significantly associated with mean annual temperature (Fig 4A and S6 Table). Of those 13,057 SNPs, only 4% were non-synonymous, while 8.7% were synonymous and 87.3%, were non-coding. These proportions are similar to those seen in the eastern transect [33] and are roughly similar to the fractions of variable sites in the dataset. Nonetheless, the very small number of non-synonymous sites showing signatures of selection suggests that selection is acting primarily on regulatory rather than on protein-coding changes. We narrowed the list of candidate loci under selection by using a more stringent q-value cut-off (q = 0.001) and by only including genes with at least two SNPs meeting this cut-off. These more stringent filters identified 311 SNPs in 95 genes (S7 Table). Among these top candidates are genes annotated to phenotypes that likely mediate responses to temperature, precipitation, and seasonality across the western transect such as osmoregulation in the gut epithelia (*Vipr1*), circadian rhythm (*Per2*), skeletal development and body size (*Bmp7*, *Bmp5*), kidney function (*Pkhd1*), metabolism & body weight (*Mc3r*), and heat sensing (*Trpm2*). All of the top candidate genes complete with functional information are listed in S7 Table.

### Parallel evolution in eastern and western populations

To address the extent of parallel genomic evolution we evaluated the overlap between LFMM outlier loci in both transects at two levels of significance: q-value<0.05 and q-value<0.001. Genetic parallelism was evaluated at the level of the gene, not the SNP. To determine whether the genetic overlap was significantly greater than expected by chance we conducted permutation tests with 100,000 replicates with replacement. At the lower stringency (q-value < 0.05), we observed 434 loci with signatures of selection in both transects, and this is significantly more than expected by chance (expected number = 339; permutation test, p-value < 0.001; S4 Fig). At the higher stringency (q-value < 0.001), we observed 16 genes with signatures of selection in both transects, also a significantly greater number than expected by chance (expected number = 5; permutation test, p-value < 0.001; S4 Fig and S8 Table). Four of these 16 genes show signatures of selection at the same SNP in the eastern and western transects, and none of these are non-synonymous mutations (S9 Table). Fourteen of these 16 genes have known functions, and of these, five have functions related to body size or fat composition (*Mc3r*, *Mtx3*), metabolism (*Galnt2*, *Zfp663*), or other aspects of thermoregulation such as temperature sensing (*Trpm2*). The observation that 31% of these genes involve traits potentially relevant to thermal adaptation suggests that much of this parallel evolution may be driven by adaptation to similar thermal gradients in eastern and western North America (Fig 1A).

**Fig 5:**
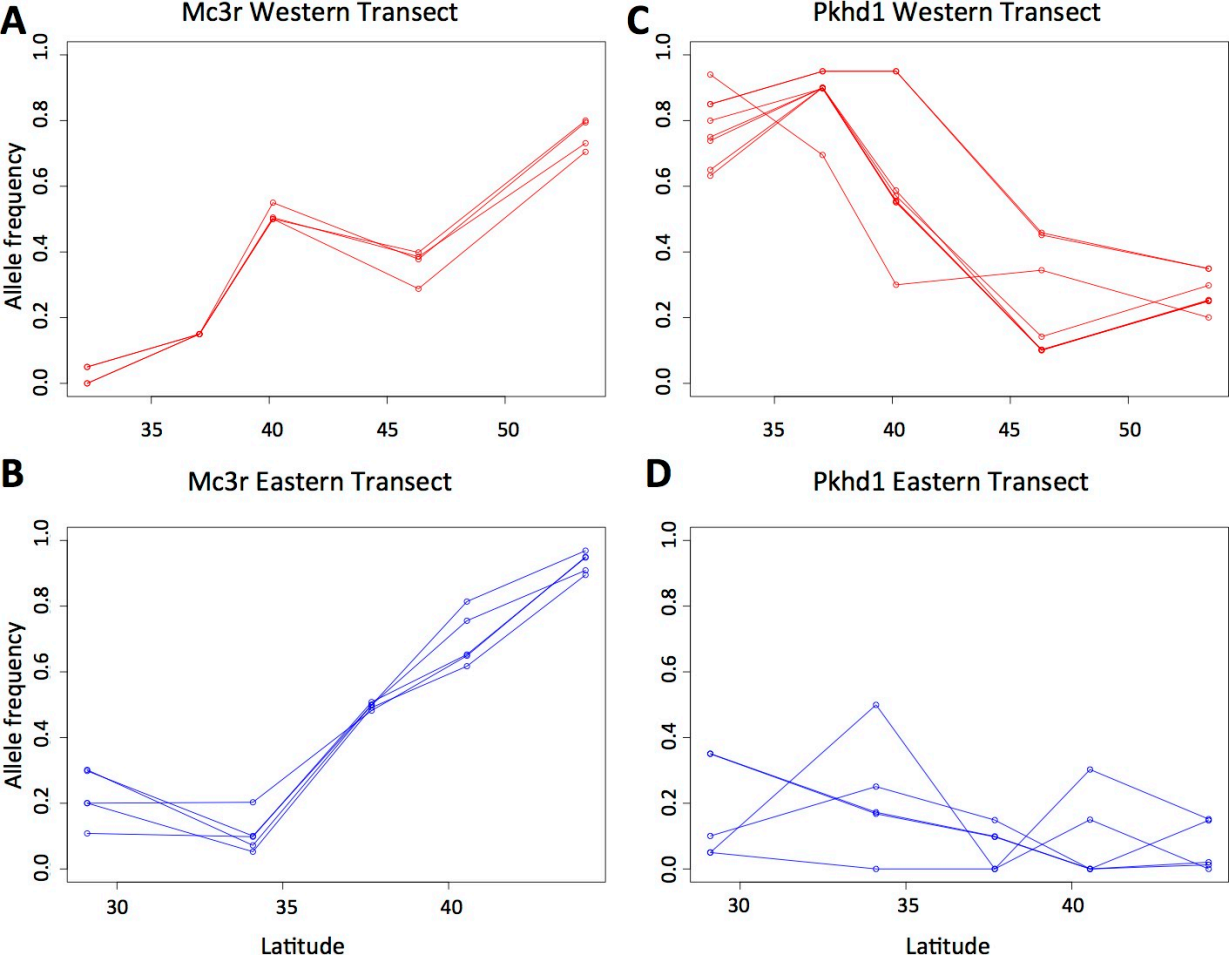
Examples of concordant and discordant clinal patterns between the eastern and western transects. Allele frequency changes at SNPs in *Mc3r* are similar in the western (A) and eastern (B) transects, while allele frequency changes at SNPs in *Pkhd1* are different in the western (C) and eastern (D) transects. *Mc3r* is believed to be involved in body size variation, while *Pkhd1* is involved in kidney function.

Two genes with similar patterns in the eastern and western transects are noteworthy for their large changes in allele frequency and known relationship to traits that are likely adaptive along latitudinal gradients. Melanocortin receptor 3 (*Mc3r*) showed differences in allele frequency of 80% in both transects (Fig 5A and 5B) and is known to be involved in feeding, metabolism, and body weight [39]. *Mc3r* knock-out mice have significantly greater fat mass and lower lean mass than wild type mice [39]. Thermo-TRP ion channel 2 (*Trpm2*) showed shifts in allele frequency of 70% in both transects and encodes a neuronal axon ion channel involved in the sensation of non-noxious heat. Expression of *Trpm2* in response to warm temperatures causes mice to behaviorally seek cooler temperatures [40]. Genetic variation in *Trpm2* may underlie adaptive variation in behavioral thermoregulation in response to increasing temperatures in southern populations.

### Divergent evolution in eastern and western populations

In addition to the greater than expected patterns of parallel genetic change, we observed that most loci show signatures of selection in only one transect. For example, a locus associated with kidney function, *Pkdh1*, showed strong patterns of clinal variation in the western transect where there is a significant cline in mean annual precipitation (Fig 5C), but *Pkdh1* did not show clinal patterns of variation in the eastern transect where there is little variation in precipitation (Fig 5D). In fact, of the top 10 candidate loci under selection (i.e. LFMM q< 0.05) in the western transect with known kidney-related functions, nine show weak or no signatures of selection in the eastern transect. Similarly, one locus showing signatures of selection in the western transect (S6 Table) that is potentially involved in coat color variation, *Adam12*, did not show signatures of selection in the eastern transect (S8 Table). This is consistent with patterns of environmental variation, with pronounced clines for soil color in the western transect but not in the eastern transect.

### Genome wide association study of body weight

To link patterns of genetic variation with known phenotypic differences in body weight, we conducted a genome wide association study (GWAS) using GEMMA [41]. Using the exome data generated here, we tested for an association between body weight and genotype at each SNP among the mice from the western transect. We used a linear mixed-model controlling for genetic relatedness and sex as covariates and a false discovery rate of 5% to control for multiple testing. We also analyzed the data from [33] in the same way to look for associations between body weight and SNPs among mice from the eastern transect, however after correcting for multiple testing there were no significant SNPs associated with body weight in eastern North America.

We found that eight SNPs in five genes were significantly associated with variation in mouse body weight in the western transect (q-value < 0.05; Fig 4B and S10 Table). All of these loci except *Lrrfip2* show signatures of selection in both western and eastern North America (LFMM q-value < 0.05). The average difference in allele frequency between the southernmost population and the northernmost population for these eight SNPs was 0.33, consistent with the idea that polygenic adaptation may be driven by modest changes in allele frequency at many genes [42]. Collectively, allelic variation at these five genes accounts for 1.82% of the phenotypic variance in mouse body weight in the western transect. Of these five genes, *Cep85*, *Cdh8*, and *Epm2aip1* have established links to body mass or metabolism in lab mice or humans (S10 Table). *Centrosomal protein 85* (*Cep85*) contains SNPs with a strong signature of selection (LFMM q-value = 0.006), and *Cep85* expression is associated with variation in female body mass index in the eastern transect [43]. Cadherin 8 (*Cdh8*) also contains SNPs with a strong signature of selection (LFMM q-value < 0.001) and has been linked to obesity and metabolic traits through QTL mapping and differential expression analysis in mice [44]. *Epm2aip1* (LFMM q-value = 0.002) is involved in glycogen metabolism [45]; inactivation of this gene in laboratory mice causes hepatic insulin resistance and resistance to age-related obesity [46]. Variation at each of these genes explains less than one percent of the variance in mouse body weight along the western transect (*Cep85* PVE = 0.46%; *Cdh8* PVE = 0.47%; *Epm2aip1* PVE = 0.45%). The functional information linking these three genes to body weight or metabolism, combined with their signatures of selection in both transects, makes them strong candidates for adaptive variation in mouse body weight.

## Discussion

We collected house mice along a latitudinal transect in western North America and compared these to house mice sampled along a similar thermal gradient in eastern North America [33]. First, we found that mice within each transect were more closely related to each other than they were to mice in the other transect. Nonetheless, lab-born progeny of mice sampled from the ends of both transects showed parallel differences in body weight and nest building behavior, suggesting that these adaptations have evolved independently in each transect. Second, genome scans identified candidate genes for environmental adaptation in the western transect and revealed that the overlap among candidate genes for each transect was more than expected by chance indicating parallel evolution occurring at the level of the genetic locus. Nevertheless, each transect also contained a number of unique candidates which may be driven by divergent environmental features in eastern and western North America. Finally, a small subset of genes showed GWAS hits for body weight in the western transect and signatures of selection in both transects. These genes are attractive candidates for linking genotype to phenotype for an adaptive quantitative trait. Below we discuss each of these issues in turn.

### Colonization history and rapid phenotypic evolution in North America

House mice have been spread around the world in association with humans [47] and likely colonized the Americas during the last few hundred years. The earliest museum records of *Mus* in the Americas date to the early 1800’s, but it is likely that house mouse populations were established before that time. In the wild, mice breed seasonally and may undergo ~2 generations per year. Thus, mice have likely been in the Americas for 400–500 generations or more. In this short evolutionary timeframe, mice have adapted to a wide range of environmental conditions.

Since house mice are an important biomedical model system, it is worth noting a number of parallels between humans and house mice in the context of adaptation in the Americas. First, the timeframe for house mice in the Americas is similar to the timeframe for humans when measured in generations. Humans colonized the Americas ~15,000 years ago [48]. With a generation time of 28 years [49], this corresponds to ~500 generations. Second, house mice are commensal and live in close association with humans. House mice are frequently found in outdoor structures such as barns, sheds, and grain storage locations where they are exposed to similar environmental pressures as native rodents (e.g. ambient temperatures, pathogens, predators). Finally, like mice, humans show evidence of adaptive differences among populations from different environments in the Americas [50,51].

Details of the colonization history of house mice in North America from their ancestral range in western Europe are mostly unknown, but several conclusions can be drawn from the available data. Levels of nucleotide diversity in North American populations are similar to those seen in Europe [52,53], suggesting that the colonization of North America was not associated with a very strong bottleneck. Patterns of genetic variation in eastern North America do not show isolation-by-distance, arguing against a single introduction from which mice dispersed [33]. The relationships of populations depicted in Fig 1 indicate that mice are grouped more by longitude than by latitude, consistent with repeated colonizations of similar latitudes in eastern and western North America. Finally, the presence of *M*. *m*. *castaneus* alleles in Tucson suggests a connection with southern California where the presence of *M*. *m*. *castaneus* alleles has previously been documented [54]. These alleles were not found in other populations in the western transect. A railroad line connects southern California with Tucson and may have provided a conduit for dispersal of mice.

We find that, despite their recent introduction, house mice in western North America are significantly differentiated in many ecologically important traits including body weight, nest building behavior, and coat color. There was significant clinal variation in dorsal coat color in the western transect, with southern mice having lighter fur than mice in the north (Fig 3E). Matching dorsal fur with background soil environments is an important anti-predator adaptation in small ground-dwelling mammals [55]. The latitudinal coat color pattern that we found matches the general transition of background soil cover from southern regions with lighter colored soils that are more sparsely vegetated to northern locations with darker soils that are more densely vegetated. Similar patterns of color variation were not evident in the eastern transect. This divergence in fur color variation between the transects is consistent with environmental differences since there is a great deal of variation in soil brightness and precipitation across the intermountain West (Fig 1), while in the East these environmental factors are fairly uniform [33].

The patterns of house mouse colonization discussed above provide a context for understanding the parallel differences in body size and nest-building behavior seen between northern and southern populations in both transects. Phenotypic measurements of fifth-generation lab-reared mice from the ends of the western transect demonstrated that there is a genetically determined difference in body weight, with significantly heavier mice in the north compared to the south (Fig 3A). These results parallel differences in body weight seen in the eastern transect [33] and are consistent with Bergmann’s rule [56]. Larger mice have lower surface area to volume ratios and therefore suffer less heat loss [57]. Therefore, heavier mice from Alberta and New York should be better able to thermoregulate during cold northern winters than mice from more southern latitudes. Bergmann’s rule has been described for both body mass and body size, although in mammals, associations with latitude are typically stronger for body mass [58]. In most cases, as in the present study, body size and body mass are strongly correlated (linear regression R^2^ = 0.57, P< 0.0001, data in S1 Table).

Nest building behavior also showed a genetically-determined difference between fifth-generation lab-reared mice from the ends of the western transect. Mice from Alberta built nests twice as large on average (12.49g) as mice from Tucson (5.58g, Fig 3B, 3C, and 3D). A similar pattern was observed in lab-reared mice from eastern North America, where New York mice built nests twice as large as mice from Florida (Fig 1C) [33]. This genetic difference in nest weight is likely adaptive since a larger nest will better insulate mice from cold winter temperatures in the north. The observation of parallel differences in body size and nest building among mice in the eastern and western transects, combined with the separate evolutionary history of the mice in each transect (Fig 1), suggests that these phenotypic differences have arisen independently, presumably as an adaptive response to novel thermal environments.

### Parallel and unique genomic changes underlie clinal adaptation

Parallel phenotypic changes in similar environments provide an opportunity to study the repeatability of evolution at the genetic level. The degree of parallelism may depend on many factors such as the relatedness of the taxa being compared, the degree of similarity between environments, and the genetic architecture of the traits being studied. A common pattern is that populations within a species exhibit greater levels of genetic parallelism than between-species comparisons, presumably because of the large proportion of shared standing genetic variation [59,60].

Much of the work on parallelism has focused on targets of selection where single genes are important, such as cardenolide resistance in diverse milkweed-feeding insects due to mutations in *ATPα* [28,61] or oxygen-binding in high-altitude birds due to mutations in *Hbb* [62]. In both cases, adaptation seems to involve a combination of parallel and unique changes. In situations involving complex traits that are highly polygenic, we expect fewer parallel changes because the target on which selection acts is so much larger. Despite this expectation, we found significantly more overlap in loci under selection between transects than expected by chance, and this pattern was robust to different significance cutoffs. This degree of overlap most likely arose as a consequence of selection having acted on the same pool of standing genetic variation in each latitudinal cline, increasing the likelihood of a similar response.

There are a number of biological and statistical reasons for expecting both shared and unique changes in house mice from eastern and western North America. While these two transects share many environmental similarities, such as a similar range in mean annual temperature, they also exhibit many differences, including considerably more variation in elevation, substrate, vegetation, and precipitation in the west compared to the east. It is likely that these differences impose distinct selective pressures in each transect, and these would be expected to lead to different genetic responses. Statistical issues also confound the easy interpretation of the proportion of shared and unique changes. The LFMM outliers undoubtedly include some false positives. Conversely, by controlling for genetic relatedness LFMM may exclude some loci that are truly under spatially varying selection, but whose variation in allele frequency is closely mirrored by population structure [63]. Additionally, there are probably genes that have responded to selection through modest changes in allele frequency [42] and these may not be detected by LFMM. Identifying polygenic signatures of adaptation remains a complex and active area of research still without perfect solutions [64–68].

Despite these caveats, we identified 16 candidate genes showing parallel adaptation to variation in mean annual temperature in both transects at a stringent false discovery rate (LFMM q-value < 0.001). Many of these 16 genes are known from studies of laboratory mouse mutants to underlie traits that are important in thermal adaptation. For example, *Mc3r* controls variation in body weight and feeding behavior in laboratory mice [39,69] and *Mc3r* shows strong clinal signatures in both transects. Another gene of note, *Trpm2* is a member of the transient receptor potential (TRP) family of thermally activated ion channels that is involved in neuronal sensing of non-noxious heat and behavioral thermoregulation in mice [40]. Activation of *Trpm2* causes mice to seek out cooler temperatures. Thermo-TRP ion channels are involved in cold and heat sensing in both invertebrates and vertebrates and have functionally diversified across species [70]. Therefore, Thermo-TRP’s are likely targets for spatially varying selection across thermal gradients. In fact, *Trmp8*, another member of this gene family, is involved in cold sensing and has been implicated in adaptation to cold in thirteen-lined ground squirrels and Syrian hamsters [71], humans [72], and woolly mammoths [73–75]. Our finding that *Trpm2* is under selection in parallel latitudinal clines of house mice suggests that both cold- and heat-sensing receptors may be important in repeated adaptation to different temperature regimes.

### Linking genotype to phenotype in an adaptive quantitative trait

Population genomic approaches are useful for detecting signatures of selection on traits that vary along ecological gradients. However, because the function of many genes remains unknown or described primarily through gene knockouts, the results of these scans can be difficult to interpret biologically. Naturally-occurring allelic variation within a gene may result in dramatically different phenotypic effects than those obtained from the inactivation of an entire locus through a knockout mutation. One way to more directly link loci under selection to phenotypic variation is to identify genome-wide associations between SNPs and adaptive traits of interest. By combining population genomic scans for selection and genome wide association studies we were able to identify a small set of genes that were significantly associated with body weight in the western transect, have established functional links to body mass in laboratory mice, and show signatures of parallel selection in the eastern and western transects: *Cdh8*, *Epm2aip1*, and *Cep85*. These genes account for a small portion of the variance in body weight, but they represent strong candidates for targets of selection related to a complex adaptive phenotype.

Many examples of the genetic basis of adaptation involve traits that are controlled by a few genes of major effect [27,28,30,76], yet most of adaptive evolution surely involves quantitative traits, where the contribution of individual genes is small. Under such situations, large changes in phenotype may be governed by modest changes in allele frequency at many loci [42]. This insight led to the development of several polygenic tests of selection based on GWAS hits for human height [64,66], although subsequent work revealed that population structure in GWAS can lead to spurious inferences [67,68]. Genome wide association studies have been performed on many traits in humans yet have rarely been applied to natural populations of other organisms. The GWAS reported in this paper was based on relatively small samples. Future studies aimed at large, well-powered GWAS on polygenic traits such as body size from a single population of wild mice would provide a means to implement more comprehensive polygenic tests of adaptation [77].

## Conclusion

This work has demonstrated rapid and parallel environmental adaptation in quantitative traits at both the phenotypic and genomic levels in house mice across North America. We found that house mice in northern populations have adapted in parallel to cold environments by becoming larger and building bigger nests compared to mice in southern populations. This adaptation appears to be largely due to regulatory changes, as opposed to protein coding changes, since very few of the signatures of selection involved non-synonymous mutations. We discovered significant overlap in the loci under selection between transects, and many of the genes identified underlie traits likely to be important in thermal adaptation, such as body size and heat-sensing. Finally, we also discovered divergent patterns of selection between the two transects at both the phenotypic and genetic levels. Color variation tracked gradients in soil color and precipitation in the western transect, but no clines for fur color were observed in the eastern transect, where less variation in soil color and precipitation is seen. Similarly, genomic patterns of variation revealed stronger evidence of selection on genes involved in kidney function in the western transect compared to the eastern transect. Together our results show that a mixture of parallel and unique changes at both the phenotypic and genetic level may be expected when closely related populations adapt to parallel latitudinal gradients.

## Materials and methods

### Ethics statement

Animals were collected and sacrificed in accordance with protocols approved by the Institutional Animal Care and Use Committee (IACUC) of the University of Arizona and the University of California, Berkeley. All wild-caught animals were collected with permits issued from the states of Arizona, Utah, and Montana in the U.S. and the province of Alberta, Canada.

### Sampling

Fifty wild *Mus musculus* were collected along a latitudinal transect in western North America from Arizona to Alberta using Sherman live traps. Ten mice were collected from each of the following locations: Tucson, AZ, St. George, UT, Provo, UT, Missoula, MT, and Edmonton, Alberta (Fig 1A and S1 Table). Each animal was caught at least 500 m from every other animal to avoid collecting relatives. Skins, skulls, and skeletons were prepared as museum specimens and deposited in the Museum of Vertebrate Zoology, University of California, Berkeley (accession numbers are given in S1 Table). Following euthanasia, fresh tissues were collected and stored in liquid nitrogen and then kept at -80°C until used for DNA extraction and sequencing.

In addition, live mice were collected from the ends of the transect, and descendants of these wild-caught mice were used to study traits in a common laboratory environment. Fourteen mice from Tucson, AZ, USA and 27 mice from Edmonton, Alberta, Canada were used to create new inbred lines. Within locations, individual collection sites were at least 500 m from each other. Lines were established from different sites, so that lines were unrelated. Animals were shipped to the University of California, Berkeley. Wild-caught animals were mated to create the first lab-reared (N1) generation. These mice were then inbred for five generations through brother-sister mating before being phenotyped for body mass and nest building as described below.

### Phenotyping field collected and live mice

For field-collected mice we measured total length, tail length, hind limb length, ear length, body weight, and testis size. Weight and length were measured in the field by a single investigator (TAS) using a 30g micro-line spring scale (Schindellegi, Switzerland) and a ruler after euthanasia and before specimen preparation. Coat color was measured on museum specimens by assessing spectral reflectance of the mid-dorsal region using a USB2000 (Ocean Optics Inc., Dunedin, FL, USA) spectrophotometer with a dual deuterium and halogen light source. Coat brightness was assessed by calculating the total area under the average reflectance curve from 300-700nm [78]. Three measurements were taken with the probe perpendicular to the specimen and were averaged for analysis. Spectral reflectance wavelengths were recorded using SPECTRASUITE (Ocean Optics Inc., Dunedin, FL, USA).

For phenotyping, laboratory mice were housed singly after weaning in static cages at 23°C with 10 hour dark and 14 hour light cycles. Mice were phenotyped for nest building behavior and body weight, traits that are known to vary clinally in mice from eastern North America [33,79]. Thirteen Tucson mice (representing four different inbred lines) and 11 Edmonton mice (representing six different inbred lines) were phenotyped. Males and females were sampled from each line except for one Tucson line for which there was only a single female available, and two Edmonton lines for which only females were available. Mice used in these assays were 154–327 days old. Nest building behavior was measured by placing 40g of cotton on top of each cage and then weighing the remaining unused cotton 24 hours later. The difference between the initial and final cotton weights was used as a measure of nest size [33]. Body weight was measured using a digital scale. To test whether nest weight was significantly different between lab reared mice from Arizona and Alberta we used a generalized linear model (GLM) implemented in R including all mice with population, age, body weight, and sex as factors. A separate generalized linear model (GLM) was run in R to test whether body mass was significantly different between lab reared mice from Arizona and Alberta, including all mice with population, age, and sex as factors.

### Exome capture, sequencing and assembly

Genomic DNA was extracted from liver of wild-caught mice using the Gentra PureGene (Qiagen Inc., Valencia, California) DNA extraction kit following the manufacturer’s fresh tissue protocol and was quantified on a Qubit 2.0 Fluorometer (Life Technologies, Foster City, CA) using the Qubit dsDNA BR Assay Kit (Life Technologies, Foster City, CA). We sheared 1 μg of genomic DNA to less than 500bp with a Biorupter (Diagenode, Denville, NJ) by sonicating the DNA for five cycles of 30 seconds on and 30 seconds off, briefly centrifuging the tubes, and then sonicating for five more cycles. Barcoded Illumina sequencing libraries were prepared using the Meyer and Kircher protocol [80]. Libraries were amplified with Phusion High-Fidelity DNA Polymerase (Thermo Scientific) for 6–8 cycles during the indexing polymerase chain reaction (PCR). Each individual sample was amplified twice in parallel and then merged to decrease PCR stochastic drift. Individually barcoded libraries were multiplexed in groups of 10 in equimolar amounts with each pool containing 1.25μg of total DNA. Exome enrichment was conducted with five captures from the SeqCap EZ Developer Library: Mouse Exome Kit (Nimblegen, Madison, WI) with slight modification to the manufacturer protocols. DNA multiplex sample pools were combined with blocking oligonucleotides and mouse COT-1 and EZ Library and incubated on a thermocycler for 72 hours at 47°C. Following hybridization, each enriched pool was split into three PCR reactions and amplified three times in parallel for 11–14 cycles and then merged. We used qPCR to determine the postcapture-enrichment efficiency. All five enriched-pooled libraries were combined together and sequenced on three lanes of an Illumina HiSeq3000 at the UC Davis Genome Center (150-bp paired end). Targeted areas include ~ 54.3 Mb of nuclear coding and UTR sequence.

For cleaning raw sequence data we followed the general protocol outlined by [81] and [82] with some modifications. Briefly, Raw fastq reads were filtered using Skewer [83] and Trimmomatic [84] to trim adapter contaminations and low quality reads. Exact PCR and/or optical duplicate reads were removed using Super-Deduper (https://github.com/dstreett/Super-Deduper). We used Bowtie2 [85] to align the resulting reads against the *Escherichia coli* genome to remove any potential bacterial contamination in the data. Overlapping paired reads were merged using Flash [86]. After cleaning, paired-end reads and merged single-end reads from each individual library were then aligned to the *Mus musculus* reference genome (GRCm38.p3) using Novoalign (http://www.novocraft.com/products/novoalign/) and we only kept reads that mapped uniquely to the reference. We used Picard (http://broadinstitute.github.io/picard/) to add read groups and GATK v3.7 [87] to perform re-alignment around indels. We then used SAMTools/bcftools [88] to generate a VCF file that contained all sites. Each site was sequenced to an average read depth of ~31X (S3 Table). The data in the VCF file were then filtered using a custom filtering program, SNPcleaner (https://github.com/tplinderoth/ngsQC) by following the protocol specified in [82]. We masked sites within 10 bp upstream and downstream of indels. We also only kept sites where at least 70% of the samples had at least 3x coverage. For most analyses, the dataset was not pruned for SNPs in close linkage. LD decays over relatively short distances in mice and rarely extends between genes [89]. Since the data are from exomes rather than whole genomes, the number of SNPs in tight association is modest. After these filters, 635K sites were used in downstream analyses.

### SNP calling

SNP and genotype calling based on fixed coverage cutoffs can result in potential bias or introduce noise in downstream population genetic analyses [90]. To take into account the statistical uncertainties around SNP/genotype calling, we called SNPs and estimated allele frequencies using an empirical Bayesian framework implemented in ANGSD with a posterior probability of 0.95 and the p-value of the likelihood ratio test of a SNP being variable to be 1e-6 [91]. For each population we only kept variants where at least 80% of the samples had data after filtering. We also eliminated sites where the minor allele frequency was less than 5%, resulting in 342,106 SNPs total.

House mice consist of three main subspecies (*M*. *m*. *domesticus* in Western Europe, *M*. *m*. *musculus* in Eastern Europe and northern Asia, and *M*. *m*. *castaneus* in southeast Asia). *M*. *m*. *domesticus* is the subspecies that is believed to have colonized most of North and South America, although there are previous reports of introgression from *M*. *m*. *castaneus* in California [36,54]. We tested for the presence of admixture with *M*. *m*. *castaneus* in each of the five populations by acquiring whole genome sequence data for *M*. *m*. *castaneus* [53]. We downloaded fastq reads of 10 *M*. *m*. *castaneus* and 10 *M*. *m*. *domesticus* specimens (see S2 Table for SRA IDs). The raw fastq reads were cleaned, aligned and re-aligned to the same *Mus musculus* reference genome using the methods described above. Admixture with *M*. *m*. *castaneus* was investigated using “NGSAdmix” [92] implemented in ANGSD which handles genotype likelihoods in a Maximum Likelihood framework. We ran the analyses considering two to five ancestral populations (K) and 5% as the minimum minor allele frequency (minMaf) cutoff. For each value of K, we performed 10 replicates and plotted the results.

We also used genetic PCA to summarize variation within and among populations and calculated pairwise *Fst*. Both *Fst* calculations and genetic principal component analyses were implemented via the ngsTools software package [93].

### Phylogenetic analyses

To investigate the evolutionary relationships among individuals and populations, we included mice from Europe (n = 5) [53], eastern North America (n = 50) [33] and western North America (n = 50; this paper). We pruned the autosomal biallelic SNPs for linkage disequilibrium with plink v1.90 [94] using non-overlapping 50 Kb windows and an r^2^ threshold of 0.5 (—indep-pairwise 50 50 0.5). Using PAUP* v4.0 [95], we estimated a neighbor joining tree and assessed node support by bootstrapping (100 repetitions). We also estimated a population tree in PAUP* using quartet assembly with random sampling of 50,000 quartets with SVDQuartets v1.0 [96] and bootstrapping to evaluate node support (100 repetitions). We defined each North American *Mus* population as an independent lineage in the tree and European *M*. *m*. *domesticus* as the outgroup.

### Environmental association

We used the Latent Factor Mixed Model (LFMM) program to perform a population genomic scan for selection and identify candidate genes underlying environmental adaptation [38]. LFMM uses a hierarchical Bayesian mixed model based on PCA residuals to account for population genetic structure while testing for significant associations between variation in allele frequency and environmental variables. We note that LFMM generally outperforms GEMMA (see below) at identifying loci under environmental selection by having a much lower false negative rate and a similar false positive rate [38]. We ran LFMM 10 times with K = 2. We chose K = 2 as the number of appropriate latent factors to use in the LFMM model because it gave the best estimate of the genomic inflation factor (λ). P-values were adjusted to control for the false discovery rate (FDR). The distribution of p-values was examined and λ was modified to obtain a flatter distribution with a peak near zero (λ = 0.9).

We acquired mean annual temperature for each of the five sampling localities using BIOCLIM [97]. We chose mean annual temperature (MAT) as the environmental variable in our LFMM analysis because MAT was the variable most closely associated with latitude and most similar between the eastern and western transects. Outlier SNPs were identified using a false discovery rate of 5% (q-value < 0.05). To identify a narrower set of candidate genes under selection we also used a more stringent cut-off of 0.1% (q-value < 0.001) and required loci to contain at least two SNPs below this cut-off. Sex chromosomes were excluded from the analyses. Outlier loci were annotated here and below using the GRCm38.75 version of the *M*. *m*. *domesticus* genome and phenotype data from Mouse Genome Informatics (MGI) (www.informatics.jax.org). MGI compiles all mouse phenotype data, the majority of which derive from studies on classical inbred strains of mice.

In natural populations of house mice, including the Tucson population studied here, linkage disequilibrium (LD) typically extends only short distances [89]. Nonetheless, outlier SNPs in this analysis may be in LD with other nearby SNPs, including some that have not been surveyed such as intronic SNPs not captured by the exome probes. Thus, the outlier SNPs may be targets of selection themselves or may be in LD with SNPs that are targets of selection. Since LD does not typically extend over multiple genes, we have annotated the genes containing outlier SNPs, although it is possible that in some cases, the target of selection is a nearby gene. The same reasoning applies to the association study (below).

### Genome wide association study

To identify genes underlying body weight we performed a genome wide association study (GWAS) using a linear mixed model approach with the program GEMMA [41]. Linear mixed model approaches have been used to successfully control for relatedness among samples and population stratification [98–101]. Input files for mapping body weight were created with the program PLINK. A total of 339,130 SNPs were used in the final mapping analysis [102]. Sex chromosomes were excluded from the analyses. We excluded pregnant females, juveniles, and individuals whose reproductive status was uncertain from this analysis. The resulting body weight input file contained SNP genotypes and phenotypes for 38 mice that had both phenotype and genotype data from the western transect. A centered kinship matrix was created in GEMMA and the linear mixed model was run with sex and kinship as covariates. The linear mixed model used in GEMMA accounts for population structure in a GWAS by calculating kinship among sampled individuals and this should reduce false positives even with our small sample size [98].

GEMMA fits a linear mixed model in the following form:

$$y=W\alpha +x\beta +u+\epsilon ;\;u∼{MVN}_{n}(0,\lambda \tau^{-1}K),\epsilon ∼MVN_{n}(0,\tau^{-1}I_{n})$$

where *y* represents a *n*-vector of qualitative traits for *n* individuals, *W* is a *n × c* matrix of covariates, α is a c-vector of the corresponding coefficients including the intercept, *x* is an n-vector of genotypes, *β* is the effect size, *u* is a vector of random effects, *ϵ* represents a vector of errors and τ^-1^ is the variance of residual errors, λ is the ratio between the two variance components, *K* is the *n* × *n* relatedness matrix, *I*_n_ is a n × n identity matrix, and MVN is the multivariate normal distribution. In this case, *y* is a vector of bodyweight for n individuals, *x* is the n by 1 vector of genotypes, and *u* is an n by 1 vector to control for relatedness and population structure, and *ϵ* represents residual errors as an n × 1 vector. We used a genome wide 5% false discovery rate (fdr) to correct for multiple testing. To calculate the percent of the phenotypic variance explained (PVE) by each significantly associated SNP with our GEMMA output we used the following equation from [103]:

$$PVE=(2\beta^{2}MAF\left(1-MAF\right))/\left(2\beta^{2}MAF\left(1-MAF\right)+{\left(se\left(\beta \right)\right)}^{2}2NMAF\left(1-MAF\right)\right)$$

where β is the effect size of a single SNP calculated using the mixed effects model in gemma, se(β) represents the standard error of the effect size, MAF is the minor allele frequency of the focal SNP, and N is equal to the sample size. To determine the effect size of an individual gene we calculated PVE for the most highly associated SNP within that locus.

We also analyzed the data from [33] in the same way to look for associations between body weight and SNPs among mice from the eastern transect. We performed GWAS on the mice from each transect separately, rather than in a combined dataset, because the combined dataset has significant population structure due to the deep divergence between transects (Figs 1B and S2). Additionally, our motivation for performing a GWAS in each transect was to ask whether the genetic basis of body weight overlapped between the transects.

### Statistical evaluation of parallel genetic evolution

To detect parallel evolution between the east and west transects we compared the loci under selection in each transect. These analyses were performed at the level of the gene, not the SNP. A gene was considered to be an outlier if it contained at least one SNP under selection. To test whether the observed overlap was greater than expected by chance we performed a permutation test using the sample function in R without replacement with 100,000 permutations. We began with candidate genes identified by LFMM using a false discovery rate of 5% (q-value < 0.05, Z-score > 2.5). We then identified the number of overlapping outlier genes between the transects. Specifically, for each permutation we randomly sampled 7407 genes, representing the western transect outliers, from the total number of genes in the *M*. *m*. *domesticus* genome (24336). We then randomly sampled 1859 genes, representing the eastern transect outliers, from the total number of genes in the genome. We then tabulated the number of genes at the intersection of these two samples. We repeated these analyses using the outliers identified with a more stringent q-value cut-off of 0.001 in each transect (Z-score > 3.1). Ninety five percent p-value confidence intervals were calculated using equations 2 & 3 in [104].

## Supporting information

S1 Table Individuals sampled, including specimen catalog numbers, exact collecting localities, reproductive data, and body measurements of 50 wild-caught *Mus musculus* from western North America. (XLSX)

S2 Table Sample information for *Mus musculus domesticus* and *M. m. castaneus* from Harr et al. 2016 [53]. (XLSX)

S3 Table Exon capture sequencing coverage statistics of data for each mouse in the western transect on an Illumina HiSeq 4000. (XLSX)

S4 Table Average nucleotide diversity (π) and average proportion of segregating sites (θ) for each of the western transect populations: Tucson, AZ, USA (TUC); St. George, UT, USA (STG); Provo, UT, USA (PRO); Missoula, MT, USA (MIS); Edmonton, Alberta, Canada (EDM). (XLSX)

S5 Table Pairwise Fst calculated using exome capture data among the western transect populations: Tucson, AZ, USA (TUC); St. George, UT, USA (STG); Provo, UT, USA (PRO); Missoula, MT, USA (MIS); Edmonton, Alberta, Canada (EDM). (XLSX)

S6 Table List of 13,057 SNPs in 4,438 genes significantly associated with variation in MAT across western North America using LFMM at the q-value < 0.05 level. The ensemble ID, chromosome, bp position, base pair change (SNP), q-value, and allele frequency in TUC, STG, PRO, MIS, and EDM populations (from left to right) are listed for each SNP along with the start and end positions, gene name, and gene description.
(XLS)

S7 Table List of 311 SNPs in 95 genes significantly associated with variation in MAT across western North America using LFMM at the q-value < 0.001 level with at least two SNPs meeting this threshold. The ensemble ID, chromosome, bp position, base pair change (SNP), q-value, and allele frequency in TUC, STG, PRO, MIS, and EDM populations (from left to right) are listed for each SNP along with the start and end positions, gene name, and gene description.
(XLS)

S8 Table A list of the top 16 genes under selection in both transects (q-values < 0.001) with their ENSMBL ID, abbreviated gene name, full gene name, and a description of their function. Genes involved in metabolic processes have been highlighted in green, while genes involved specifically in thermoregulation are highlighted in blue.
(XLSX)

S9 Table Parallel SNPs at four of the 16 genes under selection in both transects at the q-value < 0.001 significance cut-off. (XLSX)

S10 Table Top eight SNPs in five genes (FDR q-value < 0.05) associated with body weight variation in the western transect from a GWAS conducted using GEMMA. Chromosome (chr), bp position (ps), alleles, minor allele frequency (MAF), SNP effect size (beta), effect size standard error (beta se), percent of the phenotypic variance explained by each SNP calculated according to Shim et al. 2015 (SNP PVE), likelihood ratio p-value (p_lrt), gene Ensemble ID, gene name, the lowest LFMM q-value for SNPs in that gene, and a description of gene function is listed for each significant SNP in the GWAS.
(XLSX)

S1 Fig Plots of the bioclim variables mean annual temperature, temperature seasonality, isothermality, and mean annual precipitation against latitude for each house mouse population in the western transect. (TIF)

S2 Fig An estimated population tree using SVDQuartets. Bootstrap support out of a total of 100 repetitions is represented on each node.
(TIF)

S3 Fig Genetic principal components analysis (PCA) of all 100 house mice from 10 populations across Eastern and Western North America. Circles represent populations from the Western transect: AZ (cyan), St. George, UT (black), Provo, UT (red), MT (green), AB (blue). Triangles represent populations from the Eastern transect: FL (cyan), GA (black), VA (red), PA (green), VT/NH (blue). PC1 explains 14% and PC2 5% of the genetic variance.
(TIF)

S4 Fig Permuted distributions of the number of overlapping genes expected by chance in the eastern and western transects for genes showing LFMM q < 0.05 and genes showing LFMM q < 0.001. Red lines indicate the observed number in each analysis.
(TIFF)
