# Supplementary figures and images for: The genomics of rapid climatic adaptation and parallel evolution in North American house mice
Source: PLoS Genet. 2021 Apr 29;17(4):e1009495. doi: 10.1371/journal.pgen.1009495 (PMC8084166; doi:10.1371/journal.pgen.1009495)

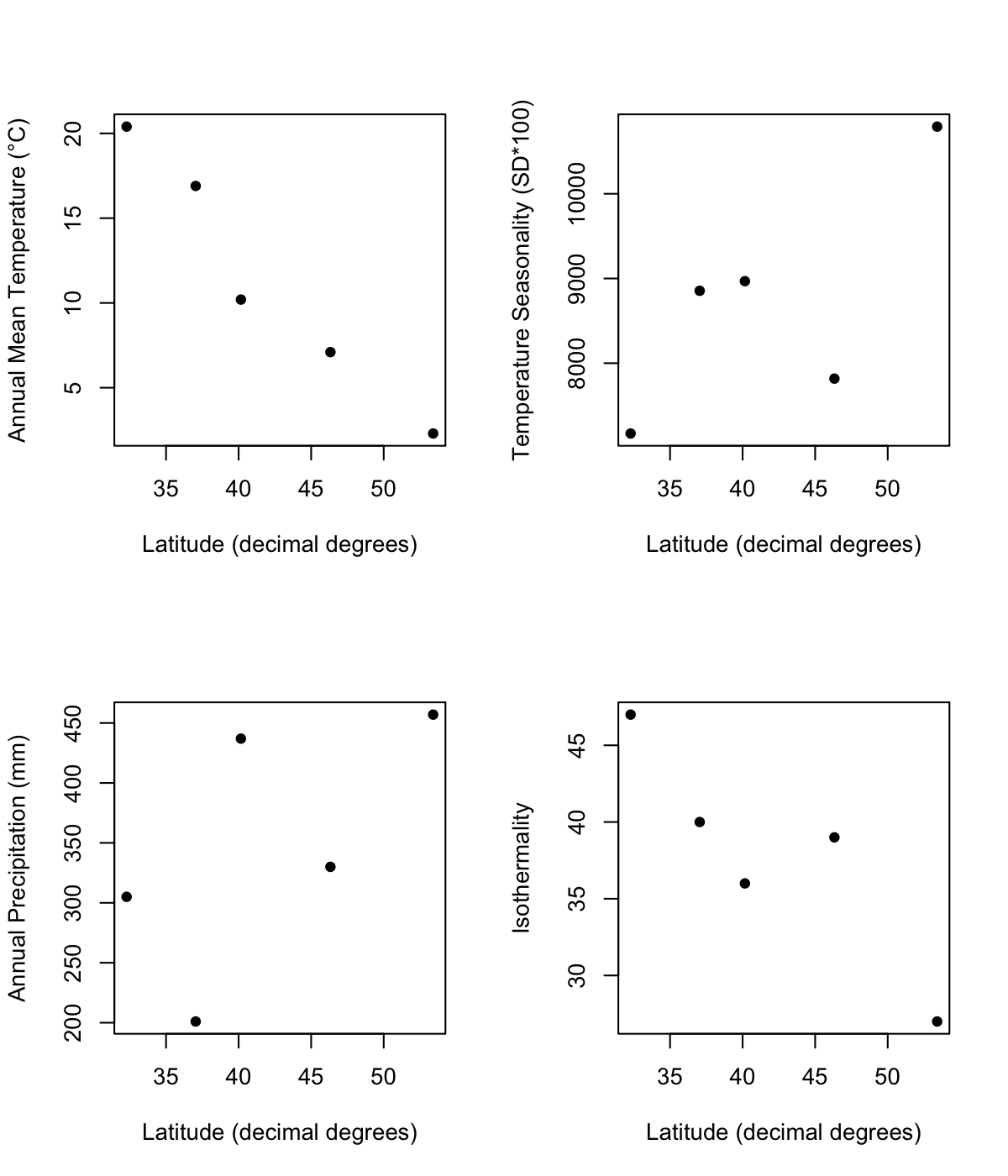

Supplement: S1 Fig — (TIF) [file pgen.1009495.s011.tif]

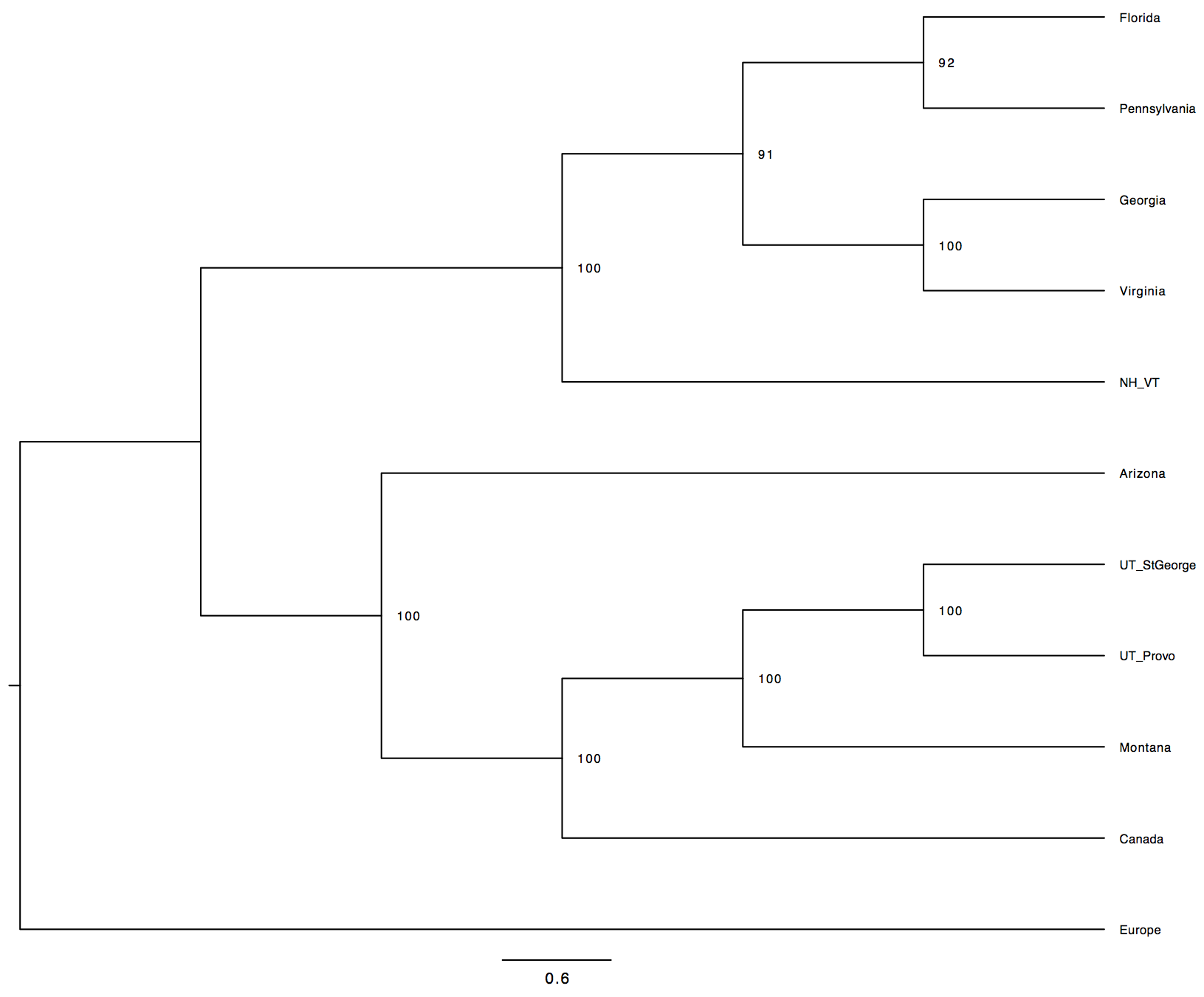

Supplement: S2 Fig — Bootstrap support out of a total of 100 repetitions is represented on each node. (TIF) [file pgen.1009495.s012.tif]

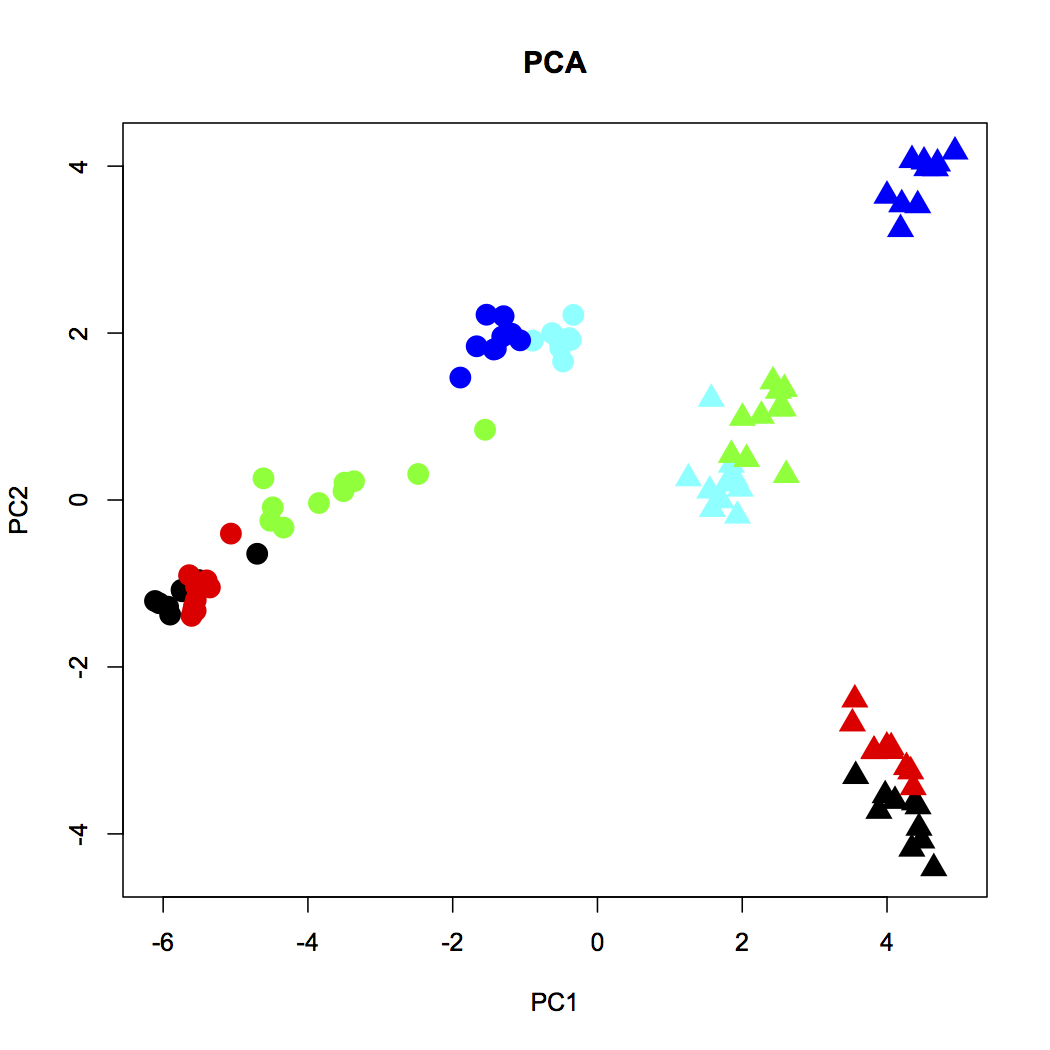

Supplement: S3 Fig — Circles represent populations from the Western transect: AZ (cyan), St. George, UT (black), Provo, UT (red), MT (green), AB (blue). Triangles represent populations from the Eastern transect: FL (cyan), GA (black), VA (red), PA (green), VT/NH (blue). PC1 explains 14% and PC2 5% of the genetic variance. (TIF) [file pgen.1009495.s013.tif]

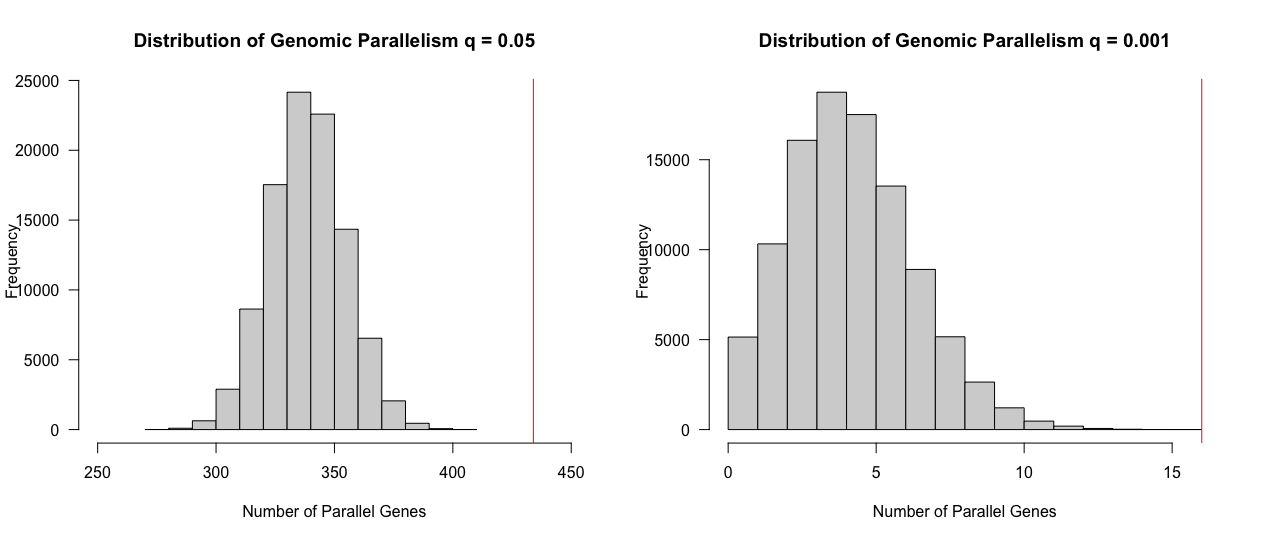

Supplement: S4 Fig — Red lines indicate the observed number in each analysis. (TIFF) [file pgen.1009495.s014.tiff]
